# Quantum Chemical Modeling of Hydrogen Bonding in Ionic Liquids

**DOI:** 10.1007/s41061-017-0142-7

**Published:** 2017-05-18

**Authors:** Patricia A. Hunt

**Affiliations:** Imperial College of Science, Technology and Medicine, London, UK

**Keywords:** Hydrogen bonding, Ionic liquids, DFT

## Abstract

Hydrogen bonding (H-bonding) is an important and very general phenomenon. H-bonding is part of the basis of life in DNA, key in controlling the properties of water and ice, and critical to modern applications such as crystal engineering, catalysis applications, pharmaceutical and agrochemical development. H-bonding also plays a significant role for many ionic liquids (IL), determining the secondary structuring and affecting key physical parameters. ILs exhibit a particularly diverse and wide range of traditional as well as non-standard forms of H-bonding, in particular the doubly ionic H-bond is important. Understanding the fundamental nature of the H-bonds that form within ILs is critical, and one way of accessing this information, that cannot be recovered by any other computational method, is through quantum chemical electronic structure calculations. However, an appropriate method and basis set must be employed, and a robust procedure for determining key structures is essential. Modern generalised solvation models have recently been extended to ILs, bringing both advantages and disadvantages. QC can provide a range of information on geometry, IR and Raman spectra, NMR spectra and at a more fundamental level through analysis of the electronic structure.

## Introduction

Hydrogen bonding (H-bonding) is an important and very general phenomenon. H-bonding is part of the basis of life in DNA, key to the environment in controlling the properties of water and ice, and critical to modern applications such as crystal engineering, catalysis applications, pharmaceutical and agrochemical development. H-bonding also plays a significant role for many ionic liquids (IL) in determining the secondary structuring and in affecting key physical parameters. ILs are being explored for an astonishingly wide range of applications; highlights include making industrial processes more sustainable, nanomaterial synthesis, solubilising metal oxides, extracting metals from ore, clean metal electrodeposition, electrolytes for batteries, biocatalysis, biomass processing, pharmaceutical and agrochemical active formulation and delivery, energetic materials, engineering fluids (lubricants, heat transfer agents) and fuels. Understanding the fundamental nature of the H-bonds that form within ILs is very important, and one way of accessing this information is through quantum chemical electronic structure (QC) calculations.

The first part of this review briefly introduces ILs and the basic concept of a H-bond. The unusual nature and wide variety of H-bonds found within ILs is emphasised and a new class of H-bond, the doubly ionic H-bond, found predominantly in ILs is introduced. QC calculations are a key route to understanding the H-bonds that form within ILs, and the importance of QC calculations in providing a benchmark for a “pure” H-bond free of external influences is discussed.

In interpreting the results and in evaluating the quality of a QC study some knowledge of the relative accuracy and utility of different QC methods is required, even for those just reading the literature and not themselves carrying out calculations. Unfortunately the literature is littered with reports of poor quality QC calculations that are substandard in terms of the methods or procedures used. The second part of this review provides an assessment of the quality of different QC approaches, suggestions are made for the minimum appropriate level of QC calculation, and for the best general methodologies to employ when studying H-bonding in ILs. QC calculations  are not the only computational methods used to study ILs; classical and ab initio molecular dynamics (MD) are also employed (more so than QC methods). It is important to be aware of the differences, and to have knowledge of the strengths and weaknesses of these other computational methods relative to QC techniques. Information from QC calculations, such as partial charges, is often used as input for MD methods; therefore, an appreciation of the limitations of both QC and MD in regard to the generation and use of partial charges is highly relevant.

To the uninitiated, establishing the correct method and basis sets, and the correct procedures to employ can be daunting. How QC calculations are carried out, the procedures used to establish key structures and an understanding of the interactions between ions is not straightforward. The correct approach for a robust study is outlined and how to identify poor procedures will be discussed. The properties that can be obtained from QC calculations and methods for interrogating the electronic structure are identified. Most QC calculations to date have been carried out on isolated ions, ion pairs and very rarely on ion pair dimers or larger clusters. However, the recent development of parameters for ILs has made the use of polarizable continuum models (PCM) possible. A broad stroke description of PCM is provided, followed by a discussion of the parameters required to model ILs using PCM. The review closes with a discussion of potential issues around using a PCM methodology.

## H-bonds and ILs

**Fig. 1:**
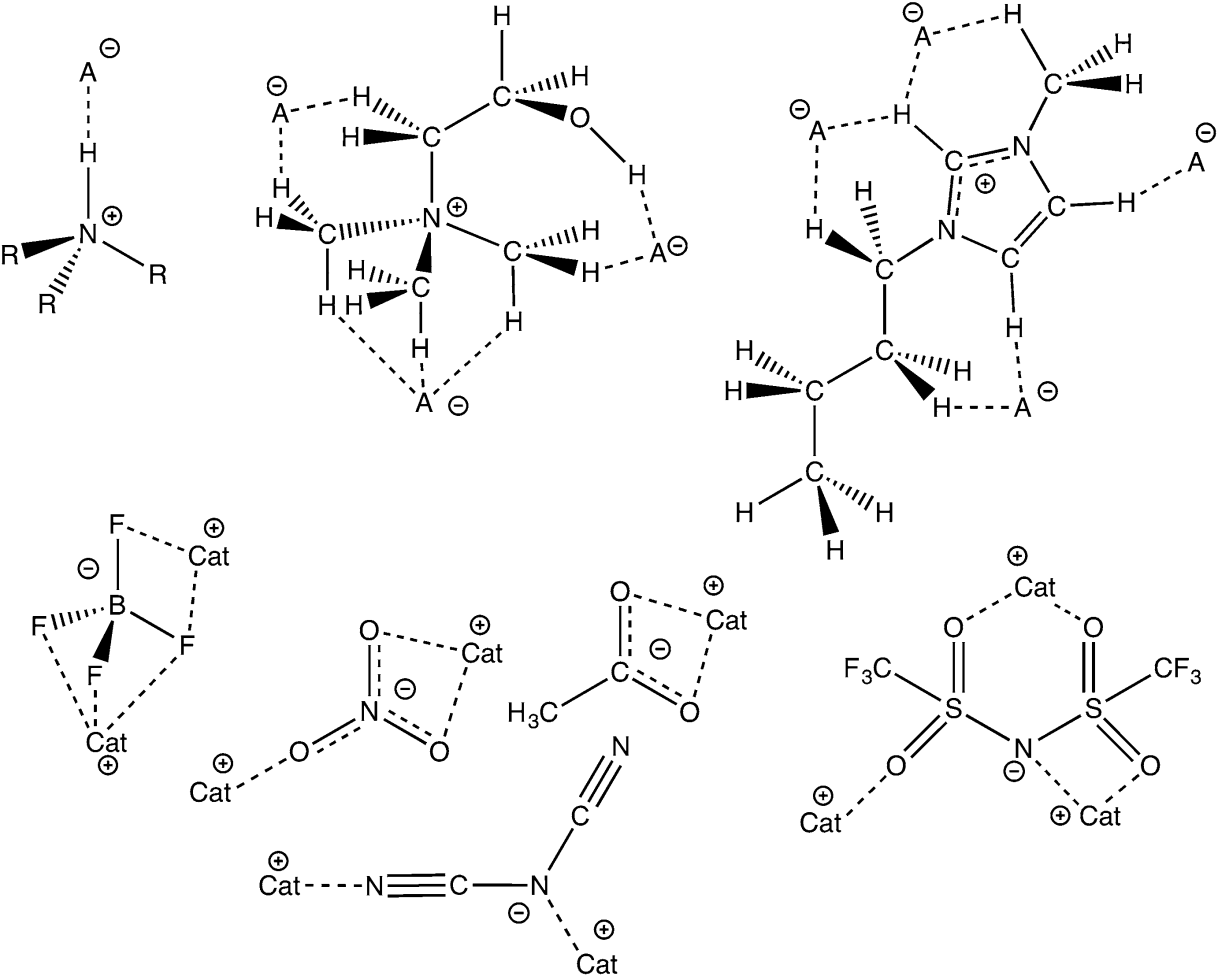
Selected H-bonding interactions

Ionic liquids (ILs) are liquids formed from structurally and chemically diverse ions. IL ions tend to be less symmetrical, larger and more diffuse than the ions found in traditional solid salts. H-bonding plays a key role in determining the chemical and physical properties of a significant number of ILs [1]. The range and versatility of ILs is in part due to the diverse range of H-bonding interactions that can occur within different ILs (Fig. 1). This extends from IL that barely exhibit H-bonding (and resemble more closely molten salts) to ILs that are formed by the transfer of a proton and exist in equilibrium with their neutral conjoiners (protic ILs).

H-bonding plays a crucial role in many of the ILs being developed for practical applications. H-bonding is key in the ability of ILs to dissolve materials that are insoluble in any other medium and for the ability of ILs to act as extraction and separation media [2–4]. For example, ILs are being used in analytical chemistry [5], in pharmaceutical applications [5, 6], in the pretreatment and dissolution of biomass components [7] and in solubilising metal oxides [8, 9]. Many reactions can be influenced by the presence of a H-bond to stabilise a key transition state. This includes biological processes, e.g. enzymic catalysis and protein stabilisation [10–12], or materials applications, e.g. proton transfer in fuel cell technologies [13, 14]. The H-bonding ability of an IL is crucial for any process that includes liquids or materials that can undertake H-bonding and in particular for any H-bonding solutes, e.g. industrial chemical reactions, solvating spectroscopic probe dyes, solvating agrochemical or pharmaceutical actives, ILs acting as electrolytes, or when the IL has small amounts of impurities or absorbed water.

H-bonding is important for supramolecular aggregation, self-assembly of biological molecules, microscopic ordering, the generation of nanoscale domains, formation of micelles and for the development of structured liquid crystal phases in crystal engineering [15–22]. Changing the H-bonding can also disrupt many of these interactions. The viscosity of an IL is heavily dependent on the H-bonding within the IL [23–25], and many potential applications in materials and engineering such as heat transfer liquids, lubricants, fuels and electrolytes depend crucially on the fluidity of the IL. In all of these applications the ability of the IL to undertake H-bonding is crucial; H-bonding plays an important but very complex role. As a result of the range of cations and anions possible, ILs can display a large range of H-bonding capability, and thus the character of the H-bonding is very system dependent [1].

Traditionally H-bonds have been defined as forming between a H-bond donor functionality (such as O–H) and a H-bond acceptor, typically with a lone pair of electrons (such as N). The H-bond forms between the small positive partial charge on the H atom and partial negative charge on the electron donor, ${\text{O}}^{\delta - } {-}{\text{H}}^{\delta } \cdots {\text{N}}^{\delta - }$. Early understanding of the H-bond focused on a single, primarily linear interaction and was considered to be essentially ionic. This view of H-bonding has now been left in the past and the identification of many new types of H-bond has led to a continuous development and blurring of the characteristics ascribed to the H-bond [26–29]. Establishing a universally accepted general definition for the H-bond is particularly challenging as opinions can differ widely. Nevertheless, general principles can be established, and IUPAC has supplied a definition with an accompanying technical report [30, 31].

It is essential to initiate any discussion on H-bonding with a description of the parameters to be employed in identifying the H-bond. We will employ the definition that a H-bonding interaction ${\text{X}}{-}{\text{H}} \cdots {\text{Y}}$ occurs if X–H acts as a proton donor to Y, where H is explicitly involved in the interaction. Nevertheless, H-bonding is not an on–off phenomenon but is a graduated scale which makes quantifying and clearly demarking H-bonding difficult. The low strength of (most) H-bonds in ILs makes them dynamic, able to form and break at room temperature. The characteristics and features of H-bonding in ILs cover an extremely wide and diverse range, and are not well understood [1, 32].

The commonly recognised *ionic H*-*bond* is one between a neutral molecule and a charged ion. However, the H-bond in an IL is between two ions, and this *doubly ionic H*-*bond* has not been recognised in the H-bonding community for the unique (type) but very common (in ILs) case it really constitutes. The characteristics of H-bonds within ILs represent a new area of development for H-bonding which currently requires further exploration [1]. The established guidelines for H-bonding have been developed primarily on the basis of traditional H-bonds, without reference to the possibility of a large range of multiple and strong doubly ionic H-bonds as found in ILs. An understanding of the doubly ionic H-bond requires reassessment of these boundaries. Many specialised definitions, based on particular properties, have been developed for the more traditional H-bond. For example, geometric distances and angles, IR spectroscopy, NMR, association energy and topological analysis of the electron density [33]. Extreme care should be taken before simply applying these definitions outside of the assigned region of applicability (traditional H-bonds) and to the doubly ionic H-bonds found in ILs [26]. Moreover, the H-bonding in ILs is not static but fluctuates and has dynamic character; this aspect of H-bonding in ILs is only just being probed.

When IL cations contain H atoms and IL anions contain atoms with lone pairs, there is the potential to form H-bonds. In typical aprotic ILs (e.g. in 1-alkyl-2-methylimidazolium-based cations [C_n_C_1_im]^+^) the primary H-bond donor is a (non-traditional) C–H unit. Formation of the H-bond is facilitated by the positive charge on the cation. Protic ILs are formed by proton transfer from a Brønsted acid to a Brønsted base. In protic ILs the cation becomes the primary H-bond donor, and the H atom is covalently bound to the heavy atom carrying the formal charge, typically N or P. If complete proton transfer is not effected, the precursor to the “anion” is a neutral acid which can also form H-bonds with fully formed anions. In addition, anions carrying a H atom can undergo H-bonding to form anion–anion H-bonds [34]. Moreover, there is also potential for alkyl groups (in the cation or anion) to be functionalized. Typical functionalities can include groups that can form strong H-bonds such as alcohols, amines and carboxylic acids. Cations with H-bond acceptor functional groups can also H-bond together; however, in this case the substituents are often shielded from the cation charge and behave more like traditional H-bonds. There has also been recent debate on smaller systems where the H atom in question is much closer to the charged centres, where “anti-electrostatic” H-bonds are suggested to form between two like charged ions [35–37]. Deep eutectic solvents (DES) are also relevant; these are formed from IL ions but also contain a neutral H-bond donor and/or H-bond acceptor molecule (e.g. urea) in varying molar ratios. Thus, in ILs, most H-bond acceptors are halogen, oxygen or nitrogen atoms and most H-bond donors are –NH, –CH or –OH groups.

**Fig. 2:**
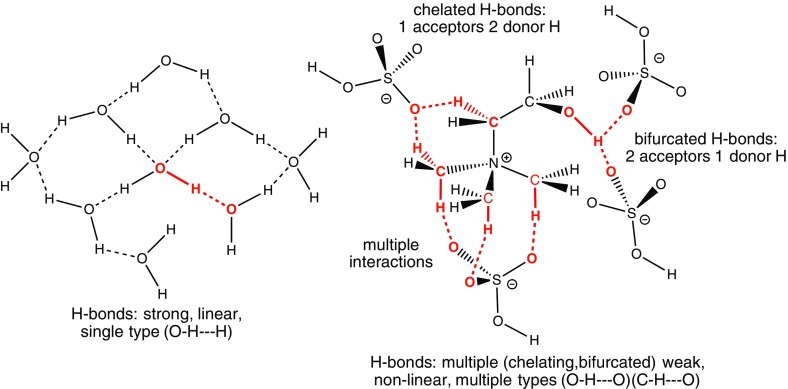
Types H-bonding interaction

Networks of H-bonds form within ILs; unlike in liquids such as water, these are not one donor–one acceptor-type H-bonds but rather exhibit bifurcated (or trifurcated) and chelating H-bonds (Fig. 2). With multiple donor and acceptor sites available H-bond networking is extensive, and it can also be highly disordered, with many “vacant” H-bonding sites. Mixtures of two or more ILs, or mixtures of ILs with traditional solvents (such as methanol) can also introduce a more diverse range of H-bonding interactions. This is particularly true of DES [38]. The characteristics of mixtures can be heavily influenced by the H-bonding interactions that take place. For example water is a ubiquitous contaminant of ILs and is capable of forming H-bonds with the IL ions; the physical properties of the resultant liquid change with the proportion of water present. Thus, the H-bonding within ILs exhibits a large range of distinctive features.

This review focuses on the QC study of H-bonding within ILs. The experimental characterization of H-bonding in ILs is a significant task and not covered here. Typically fully liquid (or even solid state) systems are studied and an “average” of all the different types and forms of H-bond is probed; studies often include a range of techniques, e.g. neutron scattering, NMR, IR, Raman, optical Kerr effect spectroscopy, or UV–vis [2, 39–43]. However, in some cases, such as in gas-phase or cryogenic ion studies, individual H-bonds can be explored [44, 45].

## Quantum Chemical Computational Modeling of H-bonds Within ILs

Ab initio QC calculations offer one way to investigate H-bonding within ILs. The computational study of traditional H-bonds through ab initio gas-phase methods is well developed and provides structural, electronic, vibrational and NMR properties. Gas-phase, or more accurately isolated molecule, calculations on ion pairs or small clusters generate very accurate association energies, electron density maps and molecular orbitals (MOs). Further analysis can be carried out on the orbital make-up (NBO [46, 47]) on the electron density (AIM [48], NCIPlot [49]) and on the electrostatic potential. With the advent of the SMD descriptors for ILs [50], the effects of a generalised solvent environment on the H-bond can also be explored. The energy associated with internal molecular rearrangements such as relative cation–anion positioning and torsional rotation in alkyl chains can also be obtained. Other computational methods such as classical MD and ab initio MD provide complementary information and have been extensively applied to ILs providing important contributions [51–55]. The focus here will be on the use of QC methods for investigating ILs and in particular H-bonding within ILs.

QC calculations provide a benchmark for a “pure” H-bond free of external influences (such as crystal packing or solvation effects). QC methods also offer a uniform mechanism for comparing H-bonds and can be used as a reference for evaluating the impact of local environmental effects (such as solvation). The fundamental isolated gas-phase doubly ionic H-bond is of substantial interest and should not be overlooked in the desire to mimic the liquid environment. Modern experimental techniques are capable of studying cold (mass) composition-selected clusters and analysing the H-bonding through IR and Raman spectra [45, 56]. The relevance of isolated ion pairs or small clusters is also evident when the mass spectra of selected clusters are analysed [57], or when small isolated clusters or ion pairs (dilute IL) are examined within a low dielectric solvent medium [43, 58–60]. An understanding of the “pure” tightly associated ion pair of an IL can offer a starting point and significant insight into the more dynamic, multiple and looser interactions within the bulk liquid state an IL. In this context, QC ion pair studies can be considered to provide a lower bound for bond lengths because the isolated ion pairs will be contracted due to  a maximised Coulombic attraction. Simple ion pair studies can also offer insight into the effects at the liquid–gas interface where H-bonding is not saturated.

## Quantum Chemical Methods

In interpreting the results and in evaluating the quality of a QC study some knowledge of the relative accuracy and utility of different QC methods is required. In carrying out simple calculations to evaluate a system, to the uninitiated, establishing the correct method and basis sets to employ can be problematic. Moreover, results can be sensitive to the computational method and basis set, making it important to use a method for which the errors are reasonable and well known. This section includes a brief discussion of QC methods and their limitations, providing some assessment of the quality of different approaches. A suggestion is made for the best general methodologies to employ when carrying out QC calculations on ILs. A recent detailed and higher-level review of the various quantum chemical approaches to studying ILs (in general) has recently been published [61].

To obtain the best energies (those converging towards an ideal “real” value), higher-level methods should be employed; these methods require very good and thus extended basis sets. Small molecules can be treated with very high levels of theory (CISD, CASSCF/MP2, CPMCSCF). However, there is a substantial increase in computational resource as the method/basis set improves and large polarizable ions, such as those found in ILs, are not easily accessible. Nevertheless, it is well established that the geometry “converges” more rapidly than the energy and little advantage will be obtained in going beyond B3LYP/6-311+G(d,p) unless there are strong dispersion or correlation effects in which case a dispersion-corrected functional or MP2-level calculation will be suitable. An often used compromise is to compute (optimise) the geometry at a lower level such as B3LYP-D3BJ/6-311+G(d,p) and then carry out a (single point) energy calculation at this geometry but now employing a higher-level method such as CCSD(T)/aug-cc-pVTZ.

Dispersion is an important consideration for ILs; ILs contain highly polarisable species and negative ions which can undergo closed shell interactions. Alkyl–alkyl associations, weak H-bonding, π-stacking, and anion–π interactions are all important. Thus for ILs, B3LYP-D3BJ (or an alternative dispersion-corrected functional) is the minimum good level that should be employed. Standard (hybrid) density functional theory (DFT) methods can under bind stable minima up to ≈30 kJ/mol (≈5% of the interaction energy) [62]. Dispersion effects can be significant, changing the relative geometry and orientation of the constituent ions within an IL; thus to obtain accurate results geometries must be computed with a dispersion-corrected method, and this is particularly important for the top conformers of imidazolium-based ILs [63, 64]. It is no surprise that when comparing across a range of typical IL anions, the dispersion contributions increase for more diffuse molecular anions; corrections are in the range of 15–25 kJ/mol [65]. Early studies on simple ion pairs were unable to evaluate the effects of π-stacking; however, a recent study on [C_1_C_4_im]_2_Cl_2_ ion pair dimers has established the importance of including dispersion corrections to correctly recover π-interactions and the relative energy ordering between π-stacked and non-π-stacked structures [63]. More concerning is a result from the same study which showed that different dispersion corrections can shift relative energies in opposing directions [63]. In addition, as the size of the alkyl groups on the ions increases, or as the number of ion pairs considered increases, dispersion contributions will increase. Recent studies employing dispersion-corrected functionals indicate that there may be some overenhancement of dispersion effects [66, 67]. The best approach therefore is to test a small number of different functionals and dispersion corrections and to be aware of the errors involved.

One need not be particularly attached to the B3LYP functional or the dispersion corrections of Grimme; there are a variety of exchange potential-incorporating functionals of similar accuracy, revPBE-D3, M06-2X or ωB97X-D [68, 69]. These dispersion-corrected functionals not only differ in the form of the DFT functional (PBE, M06, B97, B3LYP) but also in the methodology of the dispersion correction. D, D2, D3 and the more recent implementation D3BJ provide increasingly complex and more accurate ad hoc corrections based on the known functional form for classical dispersion [69–71]. The M06 suite of functionals are highly parametrised using a wide range of data including specifically noncovalent interactions such as H-bonding, charge-transfer complexes, dipole interaction complexes, weak interaction complexes and π–π stacking. The ωB97X-D functionals are range-separated functionals where the self-interaction error (which is largest at long range) is corrected by including more exact HF exchange at long range; *ω* is the range parameter controlling the switchover point [72, 73]. Thus, in a robust study all of these functionals will be tested on a limited set of structures; if the results are all very similar, one functional can then be selected to carry out all subsequent calculations with reasonable confidence.

Typically Pople basis sets [such as 6-311+G(d,p)] are less robust than Dunning-Huzinaga basis sets (such as aug-cc-pVTZ), but cost substantially less in time and resource. Generally employing a basis set beyond aug-cc-pVTZ is prohibitively expensive. Where there are polarizable anions a flexible basis set is needed; this is achieved by increasing the number and reducing the contraction of basis functions, and is indicated by the 311 in Pople and VTZ in Dunning-Huzinaga basis sets. For diffuse anions and aromatic cations diffuse functions are required; this is indicated by the “+” in Pople or the “aug” in Dunning-Huzinaga basis sets, these recover the movement of the loosely bound electron, well away from the atomic core. However, diffuse functions can make optimisation difficult, moreover diffuse functions can introduce spurious errors into population analysis. Where there is H-bonding diffuse and polarization functions are required in the basis set. Polarisation functions are indicated by the (d,p) in Pople and the “p” in Dunning-Huzinaga basis sets; these allow the sAO electron of H and the lone pairs of the H-bond acceptor to polarize towards each other, thus forming the H-bond. Basis sets must be balanced; having a very good basis set on one atom and a poor basis set on other atoms introduces errors which should be avoided. In general, for main group elements in the first and second row of the periodic table there is little advantage, and some disadvantage, in adding f-functions (e.g. in having (2df,p) in Pople notation) as the basis set becomes unbalanced. Those who specialise in H-bonding (as opposed to ILs) typically employ methods such as MP2/aug-cc-pVDZ and e.g. M06-2X/6-31+G(d,p) for larger systems where MP2 methods are too expensive [74]. As a baseline the minimum level for computationally studying normal H-bonding is B3LYP/6-31+G(d,p), where both the polarization and diffuse functions are important [75].

Population analysis methods tend to work best with medium-level basis sets; population analysis undertaken with highly extended basis sets or basis sets with more than one diffuse function per atom should be checked carefully. Too many diffuse functions cause small overlapping contributions that create instabilities and impact badly on the partition of the electron density. Large basis sets are required to obtain accurate energies; however, they can perform poorly when employed for population analysis. The self-interaction error of DFT methods generates overdelocalisation; in ILs this appears as a delocalisation of the MOs of the cation onto the anion, effectively transferring charge from the anion to the cation. The self-interaction error can be alleviated by including some HF exchange. Hybrid density functionals with a larger (>30%) HF exchange have been recommended [62].

Very recently a substantial study, focused specifically on non-covalent interactions, was carried out over a number of geometry and energy-based data sets. Non-equilibrium geometries, system size and computational time were explicitly evaluated. The functionals B3LYP-D3, M06-2X and ωB97X-D all performed well [68]. Detailed evaluations of methods and basis sets have been undertaken for a range of ILs and have shown the expected trend of accuracy for ion pair association energies; DFT methods not including HF exchange or dispersion perform poorly (≈15–30 kJ/mol), hybrid-DFT methods that include some HF exchange are better (≈15–20 kJ/mol) and methods that include HF exchange and dispersion corrections reach qualitative accuracy (≈5–10 kJ/mol). These data have been evaluated for frozen geometries optimised at the B3LYP/6-31+G(d) or MP2/6-31+G(d,p) level [76, 77]. A more definitive analysis would optimise each structure at the level to be tested.

For ILs, which have large ions and where dispersion plays an important role, B3LYP-D3 (or an alternative dispersion-corrected functional such as M06-2X or ωB97X-D) is the minimum good level that should be employed. On balance considering the performance (accuracy and time) for pure ionic liquids, H-bonding, and the ability to accurately model interactions with neutral solutes, or mixtures with molecular solvents, M06-2X may be, by a slight margin, the better choice. Because calculations must include aromatic π-density, anions and H-bonding a large basis set, which includes polarisation (to model electron movement particularly for the H atoms) and diffuse functions (to model anions correctly and to recover longer range H-bonding interactions properly) the minimum basis set is 6-311+G(d,p). At the quantitative level geometries should be optimised at the MP2/aug-cc-pVTZ level, but this can be very expensive. To carry out calculations at higher levels is extremely expensive; however, a single point energy at the CCSD(T)/aug-cc-pVTZ level is desirable. More usually computational limitations may require that CCSD(T)/aug-cc-pVDZ be employed and then there is a balance between the MP2/aug-cc-pVTZ calculation with the low method but better basis set and the CCSD(T)/aug-cc-pVDZ calculation with the better method but lower basis set.

To accurately assess the energy of a normal H-bond, it has been thought that basis set superposition error (BSSE) corrections must be applied; however, more recently the use of BSSE corrections has been questioned [78]. In general, for a high level of accuracy zero point energy (ZPE) corrections should be applied. For IL ion pairs BSSE and ZPE often lead to sizable absolute corrections, and this is particularly true when employing the MP2 method with a double-zeta basis set. When comparing different IL conformers the difference in BSSE and ZPE between the conformers is much less [63].

For ab initio methods computational resources are a major limiting factor [79]. These methods are expensive particularly when applied  to larger clusters; however, there are schemes which can simplify the computational cost. Recently a number of techniques have been applied to recover the local environmental effects within the context of ab initio methods [80–82]. However, these methods are relatively new and, while they offer promising advances, they are not well tested.

## Comparison with Other Computational Methods

Ab initio MD computes the wavefunction but within a periodically repeating boundary that mimics the liquid environment. Thus, ab initio MD includes some wavefunction and density information and some dynamics information and is a particularly powerful tool in this respect. The electron density can polarise and bond making and breaking can be studied; these are particularly important in terms of the dynamic nature of H-bonding. Ab initio MD can also provide highly relevant information on the two-dimensional space of H-bond distances (anion-H vs cation-H) [83].

From a QC perspective the disadvantage of ab initio MD is that the electronic structure cannot be computed to a high level (and typically is evaluated without HF exchange). While periodic codes have started to include HF exchange, these calculations are extremely expensive, and many codes simply do not have MP2 methods implemented. Many earlier simulation studies on ILs, state-of-the-art at the time, use non-hybrid functionals and do not include a dispersion correction, particularly ab initio MD studies. As a result of the expense ab initio MD studies are generally limited to a small number of ion pairs and the simulation “box” is necessarily small. This means there can be “image” effects as a molecule in one box is able to interact with its image in the next box. Simulations of this size cannot adequately recover nanoscale structuring, and they cannot be run for long enough to ensure phase space has been completely sampled, a particular issue for ILs which are very viscous. This also means that the initial state (derived from a classical MD simulation) may have a larger than expected impact on the ab initio simulation. The combined (electronic + periodic) nature of ab initio MD offers significant advantages, but also generates disadvantages. Thus, the best approach is to be aware of the limitations and errors inherent in each method and, where possible, combine information from a range of techniques.

The evolution of more accurate approaches which include hybrid functionals and dispersion corrections does not render earlier studies obsolete, rather the inherent small errors must be recognised when interpreting data. Earlier studies provide a valuable reference point, because we can now evaluate the effects of dispersion and/or HF exchange on the structures and dynamics within ILs. For strong H-bonds the ionic or covalent components will dominate the dispersion and exchange, and traditional ab initio methods (and even classical MD) will describe the H-bonding well; however, when H-bonds are weak or when accurate energies are required calculations must employ MP2 or the latest dispersion-corrected DFT methods, with a functional that includes a significant proportion of exchange.

Our purpose is not to discuss classical methods in any detail; however, an appreciation of the limitations and additional information classical simulation can provide is useful to place QC methods in context. Classical MD simulations recover bulk properties and macroscopic quantities such as diffusion coefficients, conductivity, viscosity and melting point. The animated trajectories obtained from MD also provide a visual interpretation of the motions occurring within the liquid, and these can be surprisingly informative. The distribution of different structures within the liquid environment is recovered. Classical MD methods capture long-range structuring within the liquids and model the nano-structured domains that exist within ILs. Classical MD also allows us to examine a key aspect of H-bonding—the dynamics. The mechanism, rate of bond breaking and reforming and the lifetime of a H-bond all contribute to our understanding of H-bonding within ILs.

The disadvantage of classical MD methods is that the potential needs to be carefully constructed to recover key chemistry, and this is particularly difficult in the case of H-bonding [54]. The problems with generating a good H-bonding water potential have shown that creating a good all round classical potential is extremely difficult [84–86]. MD simulations are only as good as the potential employed and thus can return artificial data [87]. The focus in creating MD potentials to study ILs has typically been to generate good structural averages and macroscopic properties, and not to accurately capture the H-bonding.

The first problem is that many classical MD simulations are based on a “static” electronic distribution of fixed atomic charges. While the H-bonding in IL is dominated by the ionic component, for an accurate description covalent and polarization contributions need to be recovered. Static point charges cannot represent local polarization or electron transfer within a liquid. For example, asymmetric H-bonding in clusters of [N_2111_][NO_3_] observed at the QC level has not been reproduced in MD simulations, and this was ascribed to the highly symmetric force field employed for the NO_3_^−^ anion which could not adequately reproduce the polarization occurring when only two of the O atoms within NO_3_^−^ formed a H-bond. Thus, the use of symmetric force fields can be expected to overstructure an IL [88]. This is a severe and critical limiting factor when studying H-bonding.

Potential functions and charges are fit to reproduce macroscopic thermodynamic parameters and not local interactions, and this means that atomic charges can vary substantially between models [89]. As ILs are composed of ions, the precise charge on an ion is important. This may not be such a problem at “long range” where the electrostatic potential is accurately represented; however, at H-bonding distances, the impact of local charges is significant particularly when the H-bonding is predominantly ionic. The local “charge” on an atom shifts dynamically, and the ability of an “average” charge to recover key features will depend on the full range of charge oscillations and on their frequency. Small rapid oscillations are likely well recovered by an average quantity; however, slower large oscillations are much less likely to be well represented by an average charge. It has been shown that the partial charges employed for an IL potential can strongly influence the H-bonding within the resulting simulation [87].

There has been recent debate within the community regarding the extent to which charge transfer should be accommodated within the potential [90]. Charge transfer occurs via the overlap of orbitals (which decay exponentially), and this is a close-range phenomenon and relative to the liquid environment is overestimated by gas-phase QC calculations. Moreover in a liquid environment as the H-bonding changes, the amount of charge transfer will change also. Thus, it is inappropriate to use the charge transfer predicted by the tightly bound gas-phase ion pairs for liquid-phase simulations. A slightly reduced charge on of all the ions can potentially recover some of the average effects [90]. The development of polarisable force fields able to recover the “sloshing” of charge within an ion is highly desirable, particularly if H-bonding is to be well described. It is clear that the MD representation of H-bonding in ILs needs to be improved; however, the mechanism by which this can best be achieved is less clear [90, 91].

Each computational method has strengths and weaknesses and it is the combined information from these methods which has the greatest power to offer understanding and insight. Even within each methodology there is a trade-off between complexity of the method and computation expense, e.g. the complexity of the potential vs duration of the trajectory in MD methods or the amount of exchange and correlation included in ab initio methods. Ab initio and MD methods are most informative when they are applied to exactly the same IL, and the information obtained from each can be overlapped to check each against the other; used in combination both methodologies can extend our understanding.

## Procedures for Carrying Out Calculations

The appropriate method and basis set to select when studying ILs have been discussed. How the relevant ion pair structures (for in-depth analysis) are arrived at is important. In traditional QC studies one finds the lowest energy structure and ignores higher energy conformers. This is not an appropriate procedure when there are a range of different structures with very similar energies; all the low energy structures should be interrogated. IL cation–anion pairs almost always exhibit a range of low energy structures. In each case the ion pair conformers exhibit very different H-bonding motifs. It is unfortunate that many studies compute and analyse only a single conformer.

Putting together the lowest energy structures computed for the individual isolated cation and anion (separately) will not necessarily lead to the lowest energy ion pair [92]. This has been shown to be the case for clusters as small as the [C_1_C_1_im]Cl ion pair dimers [63]. Simply placing the anion close to the C^2^–H group of the imidazolium cation and optimising a single structure will not necessarily lead to the lowest energy structure, or provide any indication that there are a significant number of other potential structures with similar energies. A good study will need to sample a full range of both low and high energy structures to obtain a balanced picture of the H-bonding and conformers available. In terms of ILs it is known that the anion can occupy different positions and orientations around the cation. For example, the energy difference between the front (C^2^–H-based interaction) and top conformers (anion above the ring) of the imidazolium ILs can be less than 5 kJ/mol [92]. Rotation of the alkyl chain can also lead to multiple minima, some of which will be stabilized more by the presence of the anion than others [92–94]. For example, the* all*-*trans* alkyl chain of [C_4_C_1_im]^+^ is not the most stable in the ion pair [C_4_C_1_im]Cl or within a larger cluster [92]. Thus, the energy associated with internal molecular rearrangements such as torsional rotation in alkyl chains should be ascertained. The position of the alkyl chains effects which H atoms are accessible and most favourable for H-bonding.

Because 5–10 kJ/mol is the “ballpark” level of accuracy for DFT methods, it is essential that all structures within 10 kJ/mol of the lowest energy conformer are considered. Such structures are more properly thought of as being essentially degenerate. Moreover, the energy ordering of structures can change depending on the particular method employed [92]. In a liquid environment a range of low energy conformers will be sampled, and thus it is important to consider more than the lowest energy structure when attempting to model a liquid IL. Slightly higher energy conformers could be stabilized by other surrounding molecules in the liquid phase thus making an apparently higher energy structure in reality a favoured conformer within the liquid phase. If the study is to offer insight into a liquid environment, it is very important to consider a range of local conformers. Recently it has been established that high energy IL ion pairs (35–60 kJ/mol) can be stabilized in systems as small as ion pair dimers [63]. This makes it even more important to sample a good range of structures when considering only the ion pairs.

It is well established in the study of ion or molecular solvation that each solvent molecule added to a solvation shell increases the number of possible structural isomers; moreover, the isomers can be close in energy, separated by small thermally accessible barriers [28]. Sampling of clusters can be minimized when there is a strong energy sink, one cluster size or conformer that is significantly more stable than others. However, for ILs, this will almost certainly not be the case; there will be a large range of low energy structures with substantial geometric and H-bonding differences. ILs in this context are reminiscent of strongly H-bonding liquids, amorphous solids or glassy systems. The very large number of possible conformers means that qualitative sampling, ideally of relevant conformers, is generally imposed. The caveat that must always be applied is that the most stable or the most relevant cluster may have been missed. Hence, it is important to explain in detail the robust processes for structural sampling.

The study of ion pairs probes detailed intermolecular interactions and for the study of IL provides information that is best used in association with additional data on longer-range properties (experimental or computational). Clusters can be built up starting from ion pair dimers (two cations, two anions) to ion pair trimers and so on. Cluster calculations at the ab initio level allow for some of the longer-range effects and larger system interactions to be recovered. The more molecules that are present, the larger the calculation  and  in many cases the method and basis set must be reduced to make the calculations tractable. Thus as one aspect of the computation is improved, another must be downgraded.

Properties such as charge polarization and vibrational modes are highly dependent on the specific arrangement of ions, and examining a single conformer or cluster motif cannot give a balanced picture. Thus, it is very important that a suitable range of low energy cluster conformers are sampled. A minimum number of different clusters to sample would be in the range of 20–30 structures. How large should a cluster be? The size of cluster is more likely to be determined by the maximum computational resources available. There is some evidence that clusters of eight ion pairs may be sufficient to recover key features of the electronic character of the liquid environment [80, 95]. However, along with larger clusters, and increased numbers of cluster conformers comes an overload of information which can be difficult to process and pull into a cohesive model or description.

## Properties and Bonding Analysis

Calculations can be used to obtain structural, energetic, spectroscopic and bonding information. Structural information such as bond distances and angles comes from optimised geometries. These will be free of the strong crystal packing effects due to the ionic nature of the solids. The vibrational analysis provides information on intensity enhancement and the H-bonding red shift [23, 92]. Vibrations can be animated and the individual atomic motions viewed. Vibrational data can be linked to experimental spectra (IR and Raman). However, for ion pairs and small clusters spectroscopic matrix isolation studies offer the best experimental technique for direct comparison with computed vibrations [45, 56, 96]. The computation of NMR spectra is also possible [81, 97, 98]. Accurate calculations on electronic excitations are more difficult, but can be linked to experimental UV–vis spectra [99].

Ab initio calculations provide the means for obtaining significant fundamental insight into H-bonds through analysis of the local electrostatic potentials (ESPs), electronic wavefunction and density [89]. The local ESP at particular atoms can be computed, as can the ESP at a given iso-surface defined around the outside of the molecule [99]. Population analysis methods deliver partial charges and dipole moments. Molecular orbitals can be depicted and interpreted [100]. Changes in the electron density *ρ*(*r*) on forming a H-bond, as well as the gradient and Laplacian of *ρ*(*r*) can be examined to characterize the formed H-bond [1]. Information on bonding and delocalisation, is readily obtained.

Each method provides a new dimension for examining the H-bond, and we obtain a rich resource of information that can be combined to obtain a fuller picture. Such methods can also be more or less sensitive to the computational method and basis set used to derive the geometry, electron density or energy, and so it is important to ascertain an estimate of the variation and/or error introduced by the method and basis set [101]. Thus, the best ab-initio studies will not just examine the relevant H-bond distances but will also consider other H-bonding characteristics such as IR-vibrations, electron density distributions and orbital descriptors.

Two methods in particular stand out in the study of H-bonds—the quantum theory of atoms in molecules (AIM) method of Bader [48] and the natural bond orbital (NBO) analysis of Weinhold [46, 102]. The proper interpretation of NBO results has recently been under debate [37, 47, 103, 104]. Different population analysis methods are based on different computed properties, e.g. AIM (density), ELF [kinetic energy density and *ρ*(*r*) gradient], NBO (wavefunction) and CHelpG (ESP) are popular choices [27]. Partial charge methods recover different information (and give different results for the same IL systems) [89, 101]. Thus, although the outputs of these methods are all called “charges” they reflect different properties, particularly in the case of density versus ESP-based methods. Comparing different structures via the same methodology is appropriate. A quantitative comparison between different methods to identify a “true” method will not be sensible; however, a qualitative comparison does provide better overall understanding. Establishing correlations between the charges from one of these methods and another property is very useful; in this case a “best method” for one particular correlation may be established. However, this does not mean that the method is the best for evaluating another property or chemical descriptor. Each of these methods provides a new dimension for examining the H-bond, and a rich resource of information is obtained that can be combined to form a better overall picture of H-bonding.

## Generalised Solvation

**Fig. 3:**
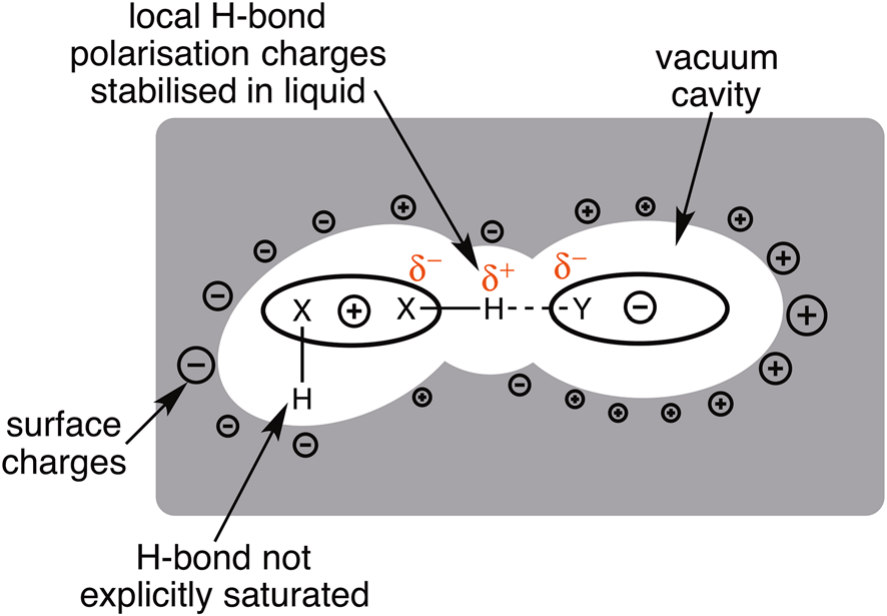
Representation of generalised solvation model

Ab initio calculations can employ a generalised solvation model (particularly polarizable continuum models, PCM) to recover average solvation effects [105, 106]. These models surround a molecule with a solvent cavity placing charges (or a continuous charge density) on the surface of the cavity to mimic the charge stabilisation of the solvent environment; the charges are modulated by the dielectric of the proposed solvent (Fig. 3). Inside the cavity the molecule still experiences a vacuum dielectric. Qualitatively, the molecule is not surrounded by solvent molecules, but the effect of the solvent environment is represented through an accumulation of opposing charge gathered on the cavity boundary. PCM methods have been traditionally developed for application to neutral organic solvents, but now extend to conducting solvents [107].

PCM models have proven extremely effective particularly for organic chemistry. However, there are known weaknesses and problems, even for organic molecules [108]. The definition of the cavity is crucial and commonly based on scaled atomic radii, which can vary with implementation [109]. Issues arise due to the use of neutral atom radii for charged ions, a rapid change of the dielectric at the cavity boundary and “escaped” charge from the exponential decay of the charge density beyond the cavity boundary (a particular issue for anions). The radii of ions have been found to be sensitive to the dielectric applied, making cations larger and anions smaller [110]. There are additional complications in applying such methods to ions, either as a solute or solvent, because of the stronger interactions. An error is introduced when the continuum model is applied too close to the ions, e.g. it has been shown that screening of (small monoatomic) ions in aqueous solution is only correct beyond 7 Å [111]. One could expect this distance to be much larger for ILs. Cavity shape can also  have a strong impact, particularly where there are diverse conformers.

A key input into PCM solvation models is the dielectric constant. The polarity of an IL is difficult to pin down; on the one hand ILs are made up of oppositely charged ions and can be expected to be extremely polar. On the other hand each ion is immediately surrounded by ions of the opposite charge which shield the central ion; moreover, these surrounding ions are well distributed, reducing polarization of the central ion in any one specific direction. Ions further away are also well shielded, reducing long-range Coulombic forces. Although formed from ions, ILs are typically thought to have a static permittivity (dielectric constant) close to that of alcohols (IL *ε* < 30, ethanol *ε* = 25) and much less than that of water (*ε* = 78) [2]. The interpretation of measurements of the static dielectric is not straightforward, and spectroscopic probe methods typically characterise ILs as having a higher dielectric than data extrapolated from dielectric relaxation spectra [112–114]. Values obtained from experimental dielectric relaxation spectra place the static dielectric permittivities of common ILs close to *ε* ≈ 12–17 [115].

The (conductor-like) COSMO-RS method has been employed successfully with ILs for some time and has recently been reviewed [116]. A number of early studies have also employed a traditional solvent such as ethanol or methanol to mimic the dielectric environment of an IL [42, 117]. The SMD method of Truhlar is based on PCM and has recently been extended to ILs solvents [50]. The SMD method computes a solute–solvent interaction but also includes terms due to cavitation, dispersion, repulsion and structure effects. The SMD method therefore requires additional data as well as  the static or zero-frequency dielectric constant (*ε*), data include;  the dynamic or optical dielectric constant [which is defined in this context as the square of the index of refraction (*n* ^2^)], the H-bond acidity (*α*), the H-bond basicity (*β*), the surface tension (*γ*), the fraction of non-H-atoms that are aromatic carbon atoms (*φ*) and the fraction of non-H-atoms that are electronegative halogen atoms (*ψ*). *φ* and *ψ* are easily determined as they depend only on the chemical formula of the IL. Kamlet-Taft measurements of α and β are also available for a large number of ILs [112]. However, where values are not available these can be readily estimated by computational methods [99]. The surface tension and refractive index for many ILs can be found on the IUPAC ionic liquids database project (IL Thermo) or from a literature search. Truhlar et al. have suggested the use of generic values as a compromise when specific data are not available [50]. A generalised solvation model specific to ILs offers the opportunity to distinguish explicit from generalised solvation effects, and an opportunity to examine (at an ab initio level) the effect of solvating one IL ion pair as solute within different ILs.

IL ions are expected to be less strongly associated in a polar dielectric medium where the surrounding charge stabilises the ions. There may be a stronger “external” impact on the ${\text{X}} \cdots {\text{Y}}$ association coordinate, and thus the doubly ionic H-bond. In this case, H-bond distances may not be a good measure of H-bond strength. For example, both neutral and ionic H-bonds have shown the same increase in bond distance with increasing polarity of a solvent medium (the solvent medium was expected to impact the ionic H-bond more strongly than the neutral H-bond) [74]. How doubly ionic H-bonds respond to a similar change in  environment has not yet been fully explored [117]. In the gas phase an ion pair is closely associated and there is significant electron transfer reducing the charge on the individual ions; the neutral state is more highly favoured. In a more polar environment an ion pair will be less closely associated, electron transfer will be reduced and the individual ions will be stabilised in a more highly charged state. The change in stabilisation/destabilisation on going from a “neutral” to a “polarized” state will effect the H-bonding proton position, and all of the X–H, ${\text{X}} \cdots {\text{Y}}$ and X–Y distances. The impact of the environment will be particularly important for (protic) systems with a low barrier to proton transfer [117, 118].

Generalised solvent-based methods cannot account for specific solute–solvent interactions, such as H-bonding. To recover these a supermolecular or cluster-based approach has often been followed for more traditional solute–solvent systems. For solutes that undertake normal H-bonding a combination of explicit solvent molecules and a surrounding continuum solvent description provides improvements over both explicitly solvated gas-phase clusters and isolated solute continuum solvation [75]. The solute and first solvation shell of solvent molecules are placed within the cavity. Placing a small cluster in a cavity has the associated issues of which cluster geometry to employ and problems associated with the definition of the solvent cavity. As the cavity surface is defined from the overlap of the atomic radii, what happens if there are “holes” where the spheres of different ions do not completely overlap within the confines of the cluster? This is a particular problem if reaction paths are explored within a PCM model.

Different cluster geometries may result in significantly different shapes and sizes of cluster cavity. How many solvent molecules should be included? Where the first solvation shell is well defined these methods can work well; however, many ions do not have strongly bound solvation shells and the number of ions in the first solvation shall can vary. Thus, the number and geometry of the solvent molecules around the solute become key parameters, as does the number of clusters sampled (e.g. from a MD trajectory). Cluster models have been tested for more traditional solute–solvent systems [119], and applied to small ions in solvents such as methanol [120]. However, in most cases the solvent molecules have been small, such as water or methanol. IL ions are large and full solvation will require a large number of these ions.

Because ILs consist of two components, the question then arises as to the treatment of the ions: should they be placed in a cavity individually, as a combined ion pair or as a small neutral or charged cluster? Ion pairs placed within a cavity will experience a strongly anisotropic polarisation due to the single explicit counter ion vs the remaining implicit counter ions. Within an explicit liquid environment each ion will be surrounded by other associating ions providing a more isotropic distribution of charge. While H-bonding solute molecules can be “saturated” by solvent molecules, as with other extended H-bond networking solvents, when more IL ions are added, increasing numbers of unsaturated H-bonds are introduced. Because IL ions undertake a large number and type of H-bonds this could be an issue with cluster/continuum solvation models applied to ILs. Weak H-bonding ILs may not be as strongly affected as protic ILs. A balance needs to be reached between the additional accuracy of including specific explicit H-bonds and the errors associated with an uneven description of the H-bonding and polarisation.

If placed within a cavity as an ion pair, explicit H-bonding is retained for one H-bond, but a generalised environment is presented for the other H-bonds; this unequal treatment of the H-bonding may lead to artefacts. However, without the explicit presence of at least one H-bond there is no charge transfer, and the ions (and the surrounding cavity surface) are “over charged”. Nevertheless, in the gas phase charge transfer is overemphasised as neutralisation of the total charge is favoured. Explicit solvation of the component ions in [C_1_C_1_im][MeSO_4_] has been shown to result in a reduced total ionic charge when compared to the gas phase [121]. As the amount of charge transferred within ILs is small 0.1–0.2 e, these effects might be considered as relatively minor.

## Conclusions

We have seen that H-bonding plays a critical role in ILs, and that ILs exhibit a particularly diverse and wide range of traditional and non-standard forms of H-bonding, in particular the doubly ionic H-bond is important. QC calculations offer one way to investigate H-bonding within ILs, providing information on geometry, IR and Raman spectra and through analysis of the electronic structure. ILs are not easy systems to study using QC techniques; however, information and insight can be obtained that cannot be recovered by any other computational method. An appropriate method of calculation combined with a robust procedure for determining key structures is required. Moreover, an appreciation of the limitations and advantages of QC techniques is important, even for those just reading the literature and not carrying out calculations. It is a combination of data and information, from a range of computational and experimental methods, which has the greatest power to offer understanding and insight.
